# Innovative Mode of Human Resource Management of University Teachers Based on Intelligent Big Data Analysis

**DOI:** 10.1155/2022/7345547

**Published:** 2022-07-01

**Authors:** Yuhong Bai

**Affiliations:** Guangzhou Huali College, Guangzhou, Guangdong 511325, China

## Abstract

In the era of knowledge economy, the competition between countries and enterprises is increasingly manifested in the competition of talents and education system. In this article, aiming at the drawbacks of the traditional HRM model of university teachers, we design and construct a management innovation model and decision-making model based on intelligent big data analysis. This article introduces DM technology. It also introduces the related knowledge of DM and the analysis and design process of HRM decision system. In this system, DM technology is used to analyze and process the existing data, predict the future situation, and provide auxiliary support for decision-making. Through simulation, the decision-making accuracy of this model can reach 95.68%, which is about 10.02% higher than other systems. It has certain practicability and reliability. This article makes a useful attempt for the application of DM technology in HRM. The research in this article is expected to play an important decision-making reference and service support for HRM of university teachers and further promote the development of HRM innovation of university teachers.

## 1. Introduction

In the modern environment, the demand for knowledge and intellectual capital in society is stronger than in any previous era, which leads to the aggravation of talent shortage. In some ways, economic growth mainly depends on the quality of labor force. Therefore, human resources are increasingly becoming strategic resources for economic and social development [1]. The competition between countries and enterprises is increasingly manifested as the competition of talents and the competition of educational system. As the quality of teaching in institution of higher learning directly determines the level of knowledge and personal quality of talents, the society has paid more attention to the management of college teachers who train senior talents [2]. However, the traditional management mode is increasingly showing many drawbacks. For example, the ideas of managers and managed people lag behind; the management mode is relatively closed and rigid; operating mechanisms such as competition mechanism, incentive mechanism, interest mechanism, responsibility mechanism, and flow mechanism are not well introduced into the management of teachers; the overall quality of teachers needs to be further improved; and teachers' resources have not been effectively allocated and developed. College teachers are the direct undertaker of college production activities and the main body and core of college human resources. The quantity, quality, and structure of teachers' resources directly determine the educational quality and school-running efficiency of institution of higher learning and then affect the national human resources and the level of economic and social development [3]. The purpose of human resource management of teachers in institution of higher learning is to mobilize the enthusiasm and creativity of every faculty member to the maximum extent and make more contributions to the school. Under the background of economic globalization, in order to realize the scientific task of human resource management in institution of higher learning, we must actively and effectively reform and innovate the human resource management of college teachers.

Relevant personnel should elevate HRM (human resource management) to the forefront of school development strategy; create a harmonious system for seeking, selecting, cultivating, knowing, loving, and employing talents; and establish a new HRM mode for modern university teachers [4]. This is critical in order to fully mobilize teachers' enthusiasm for teaching, scientific research, and work; effectively improve the quality and efficiency of school education; and promote the healthy development of higher education institutions. Various industries have accumulated a large amount of data in recent years as a result of the advancement of information technology. People's requirements for data processing technology are constantly improving due to the increasing importance of data in daily decision-making. In terms of university teacher human resource management, using intelligent big data to analyze all types of university human resource data has some practical implications for its management. We should clearly define the problems in HRM that need to be solved and recognize that DM (data mining) [5, 6] is an important step. Although the final outcome of excavation is unpredictable, the problems to be investigated should be. The requirements of DM in institutions of higher learning will continue to rise as the human management system develops [7]. As a result, improving university teachers' application research of DM technology in HRM is conducive to improving the further understanding of the theory of the HRM system in universities and promoting the improvement in the HRM system's application level. This article develops an innovative mode and decision-making model for university teachers' human resource management using intelligent big data. The following are some of its innovations: (1) This article systematically consults the theoretical and practical materials about human resource management at home and abroad, analyzes the characteristics and significance of human resource management of university teachers, and establishes the theoretical basis for research. Guided by the modern HRM theory, this article systematically analyzes the HRM situation in institution of higher learning by using DM technology “intelligently” and “automatically,” so that a large amount of data information can be effectively utilized. (2) Based on the comparative analysis of the HRM modes of Chinese and foreign university teachers, this article constructs the innovative mode and decision-making model of university teachers' HRM. The research shows that the decision-making accuracy of this model can reach 95.68%, which is about 10.02% higher than other systems. And on the basis of studying relevant models, this article finds valuable knowledge.

## 2. Related Works

The development of society requires a sufficient number of high-level human resources, and a strong country also urgently needs the growth of outstanding talents from all walks of life. Under this premise, the construction of teaching staff in institution of higher learning has been promoted to a high position, and the requirements for teachers are quite different from those before. At present, many scholars have explored the HRM of university teachers.

Ke analyzed and discussed the existing practical problems in the development and management of human resources for college teachers and put forward corresponding strategies for the development and management of human resources for college teachers [8]. Zhao et al. pointed out that HRM of college teachers is a systematic project. It not only needs to optimize the internal elements of the system as a whole, but also needs to strengthen the construction of external related systems to jointly improve the level and efficiency of management [9]. Zhou et al. outlined the application conditions of DM technology in HRM in institution of higher learning, the application of DM technology in HRM in institution of higher learning, and the scope of application of DM technology in HRM in institution of higher learning [1]. Shu outlined the meaning of human resources and HRM and analyzed the current situation of human resources and management of teachers in institution of higher learning [11]. Kessler et al. conducted a comparative analysis of the HRM models of Chinese and foreign college teachers and obtained reference and inspiration from them [12]. Haneda and I put forward a series of forward-looking and pragmatic countermeasures and solutions for strengthening the incentive mechanism of human resource development and management of college teachers [13]. Its purpose is to strengthen the management of human resources of college teachers, so as to improve the overall level of the development and management of human resources of college teachers. Sadiq et al. pointed out that due to the improvement in teachers' subject status, the special group of teachers is more suitable for people-oriented management because of their knowledge-based human resources [14]. Cerne et al., based on the relevant theories of harmonious management, put forward the idea of constructing the harmonious management model of human resources for college teachers and pointed out its operation mechanism [15]. Dhar studied the rationality and fairness of salary distribution and its impact on employees; he believes that employees not only care about the absolute amount of their own compensation, but also care about the relationship between their own pay and other' pay [16]. Sandra and Rothenberg established a dynamic programming model based on the big data background to realize the dynamic planning of enterprise human resources and proposed the initial priority table of the intelligent scheduling algorithm [17].

The related literatures are thoroughly examined in this article. Then, DM technology is used to solve the problems of talent type division and performance appraisal system in human resource management in institutions of higher learning, starting with the theory of big data analysis and combining it with the theory of human resource management in institutions of higher learning. This article proposes an innovative mode and decision-making model for managing university teachers' human resources using intelligent big data. This model employs DM technology to analyze and process existing data and forecast future outcomes and provide decision-making support. This study has a lot of practical implications for the practice of intelligent decision-making in university teacher human resource management.

## 3. Methodology

### 3.1. HRM System for College Teachers

**Figure 1:**
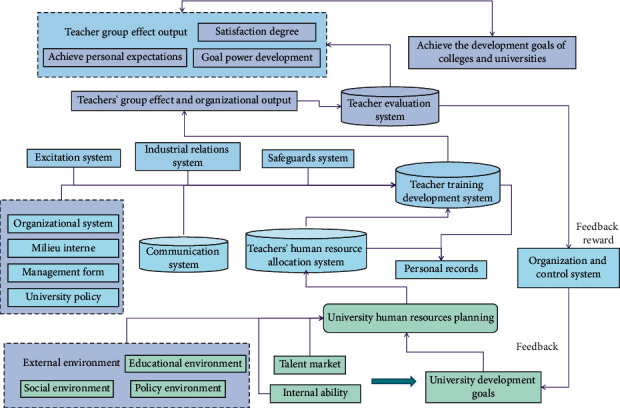
University teachers' human resource management system.

Human resources refer to the sum total of people with intellectual and physical labor ability who can promote the development of the whole society and economy within a certain range, and it is a group that can exert creative labor on the basis of labor resources [18]. It can also be said that within a certain range, it is the ability of working-age people who have been directly invested in construction and who have not yet invested in construction. Modern management science generally believes that running institution of higher learning well requires four resources: human resources, economic resources, material resources, and information resources. Among these four resources, human resources are the most important. It is the most active factor in production activities and the most important resource among all resources, which is called the first resource by economists. The macro concept of human resources is divided and measured by countries or regions. In the micro sense, the concept is divided and measured by departments, enterprises, and institutions. The most basic aspects of manpower include physical strength and intelligence. Human resource is a special and important resource for social production. Compared with other resources, this kind of resource has its own unique and distinct personality characteristics, including the times, initiative, sociality, creativity with great potential, etc. Compared with other resources, the particularity of human resources lies in the fact that it can only be developed and utilized through an effective incentive mechanism and brings visible economic value to the organization. The increasing income of human resources and its increasing economic role are not only the result of improving the quality of human resources, but also the self-enrichment characteristics of human resources. HRM refers to the use of modern scientific methods to train and allocate human resources reasonably, and at the same time to guide people's thoughts, psychology, and behaviors properly. The HRM system of college teachers constructed in this article is shown in Figure 1.

Human resources in higher education institutions cover a wide range of skills, including the total labor ability of teaching staff engaged in teaching, scientific research, management, and logistics services. The main body is that teachers demonstrate the significant social value that their teaching and educational activities, as well as scientific research and innovation activities, generate [19]. Teachers' human resource management system in higher education institutions considers all human resource management subsystems as a management engineering system. The HRM system for university teachers is built through group management. First and foremost, we must define the overall organizational goals, use these goals as the foundation for college teacher human resource management, and then create a human resource plan. Teachers' human resources refer to the sum of their knowledge, skills, attitudes, experiences, and creative ideas from the perspective of human resources. When compared to the human resources of other organizations, university teachers' human resources are unique. For example: (1) high educational level, (2) pursuing autonomy, (3) unique values, (4) strong desire to learn, and (5) strong willingness to flow. Teachers are the most precious human resources in institution of higher learning, but they are also the difficulties and emphases of human resources development in institution of higher learning. College teachers, in comparison to other human resources, have high subjective initiative, sense of accomplishment, and self-realization needs. The work is distinguished by its creativity and mobility, as well as the difficulty in monitoring and measuring the labor process. As a result, adopting a humanized and democratic management mode for teacher management is even more critical. At this stage, it is necessary to analyze the characteristics of university teachers' special human resource groups, as well as the existing problems with university teachers' human resource management, and to invent a new mode of university teachers' human resource management. The overall evaluation of university teachers' human resource management system is not only the foundation for implementing the talent rejuvenation strategy, but it is also a necessary precondition for improving the quality of university teachers' human resources and HRM efficiency. As a result, in today's environment, research on university teachers' innovative human resource management has both theoretical and practical implications. DM can identify talent patterns and rules within an organization, and thus the talent model. Simultaneously, studying DM methods, extracting valuable information, determining a reasonable human resource structure in accordance with the development goals of institutions of higher learning, and determining talent introduction and training goals are all critical.

### 3.2. Application of DM Technology

**Figure 2:**
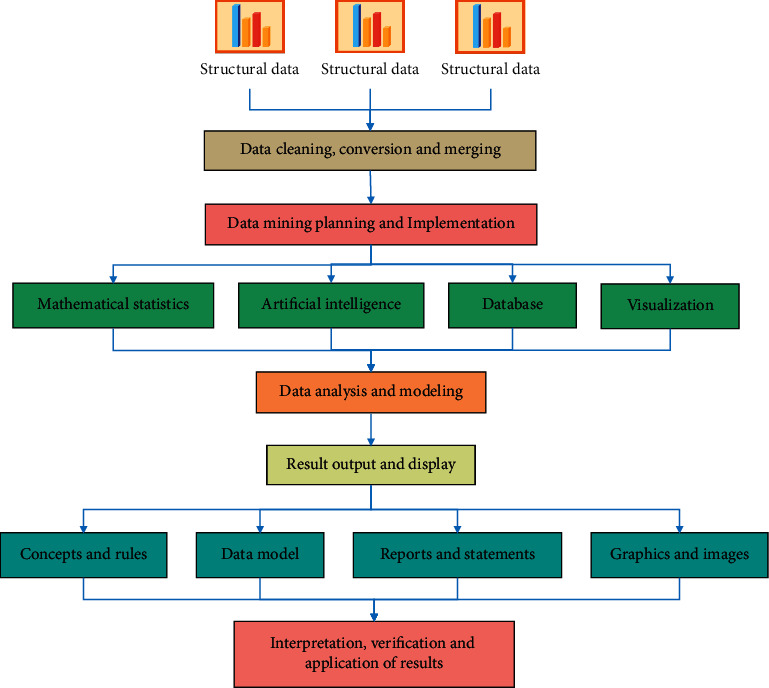
DM architecture.

Various industries have accumulated a large amount of data in recent years as a result of the development of information technology, while the database system only provides data management and simple processing functions [20]. People require a method for assisting with these complex data, locating valuable information or knowledge to aid decision-making, and reducing workload. In this case, the DM technology was proposed. DM is the process of extracting hidden information and knowledge from a large number of incomplete, noisy, fuzzy, and random practical application data that people do not know about ahead of time but could be useful. We can automatically acquire useful knowledge from fault data accumulated by human users and expand the knowledge base using DM technology. This knowledge is objective and avoids the generation of accurate knowledge because it is based on the fault data that have occurred. The problem of automatic knowledge acquisition can be solved by combining DM technology and an expert system. One of the main tasks of DM is clustering [21]. Clustering is the process of dividing data objects into subsets so that each subset is a cluster, and the objects in the cluster are similar to each other but the cluster is not similar to the objects in the cluster, that is, the cluster has the maximum similarity within the cluster but the cluster has the least similarity between clusters. The clustering model divides data into different groups which is based on the relationship between data content and data. Clustering patterns are highly random and arbitrarily distributed [22]. Association analysis uncovers two types of relationships: frequent itemsets and association rules. The frequent itemsets refer to a group of attributes that frequently appear together at the same time, whereas the association rules imply that the two attributes may have a strong relationship. Students' data are analyzed using DM technology, and a mathematical model is created using a clustering algorithm. The students are then analyzed using the correlation analysis algorithm, and an overall correlation analysis model of students is created. The DM architecture is shown in Figure 2.

To apply DM to this model, the following steps should be followed: (1) Determining the objects and targets of mining and finding out the problems clearly, (2) collecting the data, (3) carrying out the data conversion process, (4) classification and mining of data, (5) analyzing the results of classification rules, and (6) applying knowledge. The mining process is divided into two basic stages: initialization and iteration. The initial data structure is set in the initialization stage, and the decision tree is generated in the iterative process. Data cleaning of data warehouse is similar to data cleaning of DM. If the data have been cleaned when it is imported into data warehouse, it is probably unnecessary to clean it again when doing DM, and all data inconsistencies have been solved. Data extraction is the process of extracting data from the data source database of application system to the target database. Data extraction methods mainly include total extraction and incremental extraction. There are two factors to consider when choosing a mining algorithm. First, data with different properties should use algorithms related to their characteristics, and second, the user's requirements for the discovery results. If the discovered knowledge cannot meet the user's requirements, the whole discovery process needs to be returned to the discovery stage, and data should be re-selected, new data transformation methods should be adopted, new DM parameter values should be set, or even another mining algorithm should be changed. In addition, selecting and analyzing data may be the most important part of DM, even more important than algorithm selection. The reason is that DM is usually not driven by assumptions, but by data. DM can receive data and discover new associations, instead of selecting and testing variables in advance, so DM should obtain as clean data as possible and perform data profiling before trying any models. According to the requirements of DM, we should check the spelling and errors of the extracted data, check and fill the gaps in the data records, and convert the data types, which is called preprocessing.

### 3.3. Innovative Model of Teacher HRM Based on Big Data Analysis

With the enhancement of informatization in institution of higher learning, institution of higher learning now have a relatively complete information management system, and the basic data of human resource management are sufficient, which can select, preprocess, and convert human resources data and make data preparation for DM. Considering the structure of specific human resources, this article chooses the decision tree of classified knowledge discovery as the DM method. A decision tree is a tree structure similar to a flowchart, in which each internal node of the tree represents a test of an attribute, its branches represent each result of the test, and each leaf node of the tree represents a category. The top node of the tree is the root node. First, the data source is selected and the database is established. In order to prevent the repetition of data and the inconsistency of calling data, the data source is discretized before calling data, and the specific data are converted into unique letters or numbers. The quality of research data, in preparation for further analysis. And determine the type of mining operation to be performed. Data cleaning refers to the process of finding and correcting identifiable errors in data files, including checking data consistency, dealing with invalid values and missing values, etc. Generally, the principle of data cleaning is the process of reducing the database to eliminate duplicate attributes and converting the format into an acceptable standard format. Establishing a correspondence table between source data and processed data is equivalent to establishing a mapping relationship between them, which ensures that the change of one data will not affect the other, thus realizing the independence of source data and programs.

Assuming that the dataset *S* contains a set of *s* data samples, the category attribute can take *m* different values, corresponding to *m* different categories, namely:

(1) $$\begin{array}{c} C_{i},i\in \left\{1,2,3,\ldots ,m\right\}. \end{array}$$

Assuming *s*_*i*_ is the number of samples in class *C*_*i*_, the amount of information required to classify a given data object is

(2) $$\begin{array}{c} I\left(s_{1},s_{2},\ldots ,s_{m}\right)=-\sum_{i=1}^{m}p_{i}\log\left(p_{i}\right), \end{array}$$

where *p*_*i*_ is the probability that any data object belongs to category *C*_*i*_, which can be calculated by *s*_*i*_/*s*. The function of log is based on 2, because in information theory, information is encoded in bits.

Let an attribute *A* take *v* different {*a*_1_, *a*_2_,…, *a*_*v*_}. The set can be divided into *v* subsets using the attribute *A*, namely

(3) $$\begin{array}{c} \left\{S_{1},S_{2},\ldots ,S_{v}\right\}, \end{array}$$

where *S*_*j*_ contains the data samples whose public attribute value of *S* set is *a*_*j*_. If the attribute is selected as the test attribute *A*, let *s*_*ij*_ be the number of samples of the attribute *C*_*i*_ category in the subset *S*_*j*_. Then the information entropy required to divide the current sample set using attribute *A* can be calculated as follows:

(4) $$\begin{array}{c} E\left(A\right)=\sum_{j=1}^{v}\frac{s_{1j}+s_{2j}+\ldots +s_{mj}}{s}I\left(s_{ij},\ldots ,s_{mj}\right), \end{array}$$

where *s*_1*j*_+*s*_2*j*_+…+*s*_*mj*_/*s* is taken as the weight of the *i*th subset. It is divided by the sum of the sample data of attribute *A* taking *a*_*j*_ in all subsets by the total number of samples in the *S* set. The smaller the *E*(*A*) calculation result, the better the result of its subset division. Assuming that there are *n* samples in the sample set, *n* data can be obtained for a certain indicator *k* of the samples, namely

(5) $$\begin{array}{c} h_{1k}^{\prime },h_{2k}^{\prime },\ldots ,h_{nk}^{\prime }, \end{array}$$

where *h*_*nk*_′ represents the data obtained by the *i*th sample for the *k*th index. Their average is calculated as

(6) $$\begin{array}{c} h_{\bar{k}}^{'}=\frac{\left(h_{1k}^{\prime },h_{2k}^{\prime },\ldots ,h_{nk}^{\prime }\right)}{n}=\frac{\Sigma h_{ik}^{\prime }}{n},\;k=1,2,\ldots ,m. \end{array}$$

The standard deviation of these raw data is as follows:

(7) $$\begin{array}{c} S_{k}=\sqrt{\frac{\sum_{i=1}^{n}\left(h_{ik}^{\prime }-h{{}^{\prime }}_{\bar{k}}\right)}{n}}. \end{array}$$

The normalized value of each data is obtained by the following formula:

(8) $$\begin{array}{c} h_{ik}^{\prime \prime }=\left|\frac{h_{ik}^{\prime }-h_{k}^{\prime }}{S_{k}}\right|. \end{array}$$

If the extreme value standardization formula of the obtained standardized data *h*_*ik*_^″^ is not within the closed interval of [0,1], the following extreme value standardization formula is used:

(9) $$\begin{array}{c} h_{ik}=\frac{h_{ik}^{\prime \prime }-h_{\min k}^{\prime \prime }}{h_{\max k}^{\prime \prime }-h_{\min k}^{\prime \prime }}, \end{array}$$

where *h*_max*k*_^″^ and *h*_min*k*_^″^ represent the maximum and minimum values in *h*_1*k*_^″^, *h*_2*k*_^″^,…, *h*_*nk*_^″^, respectively.

DM technology can extract human resources data from a variety of databases containing information about the working conditions of the organization's employees as well as find connections and patterns among them, objectively reflecting the organization's talent pool. The most frequent set among the generated frequent sets is found and an algorithm is used to determine whether the data have any implicit relationships. Users or machines must evaluate the patterns discovered during the DM stage. Any redundant or irrelevant patterns are removed and returned to the previous stage if the mode cannot meet the user's needs. Re-selecting data, implementing new data transformation methods, adjusting parameter values, and so on are examples of such tasks. In DM, data preparation is a critical step in the knowledge discovery process, accounting for more than half of the workload. It is necessary to preprocess data, especially when DM is performed on data that contain noise, incompleteness, or even inconsistency, in order to improve the quality of DM and, ultimately, the quality of pattern knowledge acquired by DM. This article examines the human resources system's staff information database as well as the staff assessment results database. It has a relational database format, which sorts information attributes that are thought to be closely related to assessment results into data tables and deletes information attributes that are not. In order to figure out a few key variables for the DM process, the qualitative index attributes are calculated. The primary task of the implementation of the HRM plan of university teachers is to equip the organization with personnel and change the internal environment according to the requirements of the organization and then to establish the post responsibilities of all staff in each internal department. Therefore, this article establishes the following subsystems: (1) organization system, (2) incentive system, (3) communication system, (4) management relationship system and guarantee system, and (5) evaluation system. According to this HRM system, all subsystem plans and actions complement each other.

When the state is determined in the human resource scheduling of university teachers, the state transition equation can be used to show the law of this state deduction:

(10) $$\begin{array}{c} x_{p+1}=B_{p}\left(x_{p},w_{p}\left(x_{p}\right)\right),p=1,2,\ldots n. \end{array}$$

From the above equation, it is possible to further deduce the indexes that help define the whole stage process and all the back subprocesses. The formula of the index is:

(11) $$\begin{array}{c} Q_{kn}\left(x_{p},w_{p},\ldots ,x_{n+1}\right),p=1,2\ldots n. \end{array}$$

According to the state transition equation and the formula of the indicator function, in the actual scheduling of human resources, the decision set *x*_*p*_ can be determined by the characteristics of the state variable *x*_*p*_ and the decision variable *W*_*n*_(*x*_*n*_) itself, and then substituted into the state transition of equation (10). Suppose a real-time computing task requests *R*, then the sequence is

(12) $$\begin{array}{c} R=\left(e_{i}^{\text{born}},e_{i}^{\text{wait}},e_{i}^{\text{dead}},y_{i}\right), \end{array}$$

where *i* represents the real-time task number waiting to be executed, *e*_*i*_^born^ represents the generation time of the real-time task, and *e*_*i*_^wait^ represents the maximum waiting time when the task is queued for execution. This value is determined by the number of institution of higher learning, business volume, etc. Whereas *e*_*i*_^dead^ stands for task deadline and *y*_*i*_ stands for the initial priority of the task. The smaller the value, the higher the initial priority of the task. When the current time is assumed to be *s*, then the relative deadline *a*_*i*_ of the real-time task satisfies:

(13) $$\begin{array}{c} a_{i}=e_{i}^{\text{dead}}-s. \end{array}$$

From this, it can be calculated that the relative deadline *a*_*i*_ of a real-time task will gradually decrease over time, which means that its task priority will increase with time.

The association rule generation module's main task is to extract strong association rules from frequently occurring sets with the least amount of support and confidence. Because the association rules to be mined are strong association rules, and because the rules are generated by frequent itemsets, each rule meets the minimum support degree automatically, so generating association rules for frequent itemsets only requires meeting the confidence level. The decision tree induction algorithm calculates each attribute's information gain and chooses the attribute with the highest information gain as the test attribute of a given set, resulting in the branch nodes. The corresponding attributes are marked on the generated nodes, and corresponding branches of the decision tree are generated based on different values of this attribute, with each branch representing a partitioned sample subset. The generated selection tree can be used to define a rule set. A rule set is made up of a number of rules, each of which corresponds to a different path in the selection tree, which represents a link chevalier. The abstract preprocessing, frequent set mining, algorithm execution, and association rule generation processes are all intuitively displayed on the page in this article via FLASH animation. When that user enters the degree of trust and the degree of support, the text expression that corresponds to the generated strong regular formula is displayed, making it easy for the user to understand and analyze the relationship between the attribute fields.

## 4. Result Analysis and Discussion

This article studies the application of intelligent big data analysis in college teachers' human resource management, mainly using DM to solve problems in human resource management and innovating college teachers' human resource model and decision-making model. In this article, HRM is quoted from the market concept, and the management function of the HRM department in institution of higher learning is given by the internal faculty of institution of higher learning. In order to verify the feasibility of the innovative model and decision-making model of human resource management of university teachers designed and constructed in this article, this chapter conducts simulation experiments to test the performance of the model in all aspects. First, the feature selection of the five-fold cross data of the model in this article is carried out, and the experimental results are shown in Table 1.

**Figure 3:**
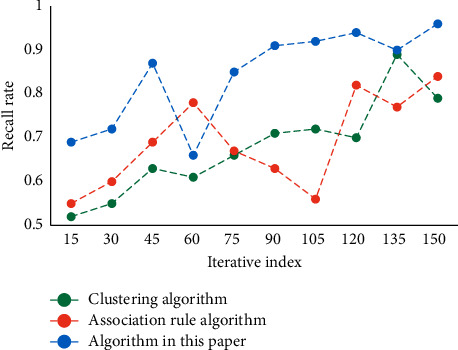
Recall results of different algorithms.

**Figure 4:**
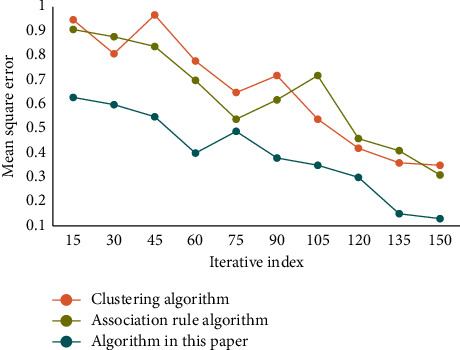
Comparison of mean square error of different algorithms.

**Figure 5:**
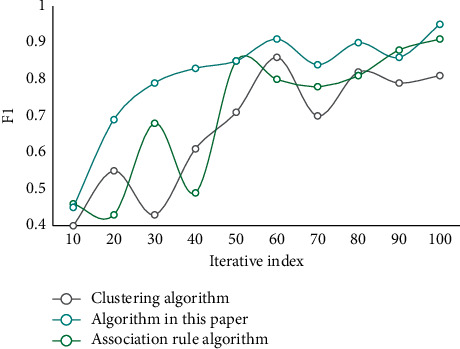
Comparison of F1 results of different algorithms.

First of all, the data should be selected, all the internal and external data information related to the HRM issues is searched, the data suitable for DM applications are selected, then the data are preprocessed, and, finally, the data are converted into an analysis model. This analysis model is built for mining algorithm. The purpose of using decision tree to classify teachers' resources is to dig out potential turnover among teachers in the management office. Then, some measures should be taken to retain some potential important teachers, so as to reduce the losses of institution of higher learning and form a stable group of teachers. To verify the performance of this algorithm, three indexes, namely recall rate, average absolute error, and F1 value, are selected for testing, and the obtained result is compared with different algorithms. The result of the recall rate is shown in Figure 3. The mean square error comparison results are shown in Figure 4. The comparison results of F1 values are shown in Figure 5.

As can be seen from the data in Figure 3, the recall rate of this algorithm has certain advantages in comparison of the recall rates of different algorithms. Its recall rate is better. As can be seen from the data in Figure 4, with the increase of iteration index, the error of each algorithm becomes smaller and smaller. However, the error of this algorithm decreases faster and its error value is lower. From the data in Figure 5, it can be seen that the F1 value of this method is always at a high level in the comparison of F1 values of different algorithms. Generally speaking, the performance of this algorithm is better. It has certain accuracy and reliability.

**Figure 6:**
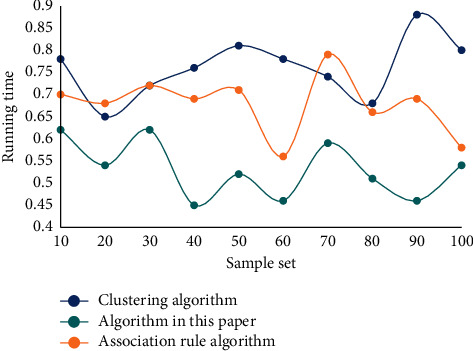
Time-consuming running of different algorithms.

In order to make the algorithm in this article more in line with the needs of human resource management of university teachers in the current era, we should give full consideration to innovating the scheduling decision algorithm of the platform and summarize the advantages and disadvantages of the algorithms that have been put into application before. Comparing the running time of different algorithms, the results are shown in Figure 6.

It can be seen that compared with the traditional algorithm, the decision algorithm proposed in this article can effectively shorten the time required to fuse and calculate the stage task data. Its operation performance and fusion effect are superior. Performance appraisal is an important task of HRM in institution of higher learning. Other work of HRM needs to be completed based on the results of performance appraisal. Therefore, the use of quantitative analysis methods to evaluate the performance appraisal system is of great significance for the effective implementation of performance appraisal. This article analyzes from two aspects: the correlation analysis between the assessment index variables, and the correlation analysis between the original index variables and the assessment index variables.  Table 2 shows the teacher metrics chosen in this article.

**Figure 7:**
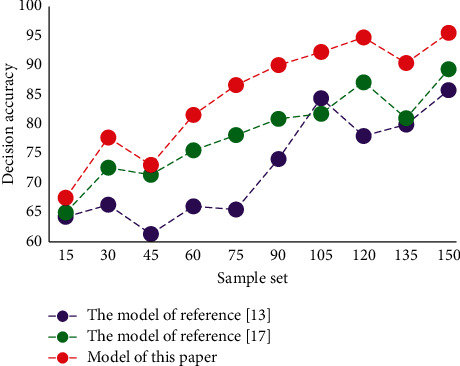
Decision accuracy results of different models.

In order to verify the feasibility of the innovative model and decision-making model of teacher human resource management constructed in this article, we make an experimental analysis of the decision-making accuracy of this model. The results obtained are compared with other models. The decision accuracy of different models is shown in Figure 7.

According to the data analysis in the figure, compared with the other two decision-making models, the decision-making accuracy of the teacher HRM decision-making model in this article is the highest. It has certain decision-making effectiveness and accuracy. In this chapter, several simulation experiments were conducted to verify the performance of the innovative model and decision-making model of university teachers' human resource management based on intelligent big data. Experimental results show that the accuracy of the system can reach 95.68%, which is about 10.02% higher than other systems. From the test results, this system performs well, is feasible, and has excellent systematic performance.

## 5. Conclusions

Human resources, in comparison to other economic resources, have a distinct purpose, subjective initiative, and unique creativity. Traditional human resource management modes and information acquisition methods limit the development of human resource management in higher education institutions. In light of the growing drawbacks of traditional HRM, this article investigates various issues in HRM in higher education institutions using intelligent big data analysis technology and related HRM and DM theories. This article develops and implements an innovative mode and decision-making model for university teachers' human resource management. This system employs DM technology to analyze and process existing data and forecast future events and provide decision-making support. The outcome of the experiment is in line with the hypothesis. This model has a decision-making accuracy of 95.68%, which is 10.02% higher than other systems. This system can significantly reduce the workload of intelligent scheduling of university teachers' human resources based on big data analysis as well as the time it takes to fuse and calculate stage task data. It is feasible and practicable in some ways. This research has yielded some results, but due to my limited knowledge and research time, there are still some areas that could be improved. The model structure will be improved in future work, as will the model performance, and a quantitative analysis scheme will be proposed to solve the HRM problems of university teachers.

## Figures and Tables

**Table 1:** Feature subset selected by quintuple cross data.

Dataset	Feature subset
Train1	4, 6, 8, 9, 11, 13, 15, 21, 26, 28
Train2	5, 6, 8, 9, 11, 13, 15, 18, 26, 28
Train3	4, 5, 6, 8, 9, 11, 13, 15, 26, 28
Train4	4, 6, 8, 9, 11, 13, 15, 16, 19, 26, 28
Train5	4, 5, 6, 8, 9, 11, 13, 15, 26, 28

**Table 2:** Selected teacher indicators.

Program	Knowledge level	Teaching skills	Teaching quality	Teaching effect
Teacher 1	4	3	3	3
Teacher 2	2	3.5	5	2
Teacher 3	3	3	5	4
Teacher 4	5	4	4	4
Teacher 5	4	4	3	3
Teacher 6	6	3	3	3
Teacher 7	5	3.5	2	2
Teacher 8	4	2	3	2
Teacher 9	5	2.5	4	3
Teacher 10	3	4	5	4

## Data Availability

The data used to support the findings of this study are available from the author upon request.
